# Pseudohypoaldosteronism in a neonate presenting as life-threatening arrhythmia

**DOI:** 10.1530/EDM-13-0077

**Published:** 2014-03-01

**Authors:** Sudeep K Rajpoot, Carlos Maggi, Amrit Bhangoo

**Affiliations:** College of Medicine, American University of AntiguaAntigua; 1 Pediatric Intensive Care, Miller Children's HospitalLong Beach, CaliforniaUSA; 2 Children Hospital of Orange County1201 W. La Veta Ave, Orange, California 92868USA

## Abstract

Neonatal hyperkalemia and hyponatremia are medical conditions that require an emergent diagnosis and treatment to avoid morbidity and mortality. Here, we describe the case of a 10-day-old female baby presenting with life-threatening hyperkalemia, hyponatremia, and metabolic acidosis diagnosed as autosomal dominant pseudohypoaldosteronism type 1 (PHA1). This report aims to recognize that PHA1 may present with a life-threatening arrhythmia due to severe hyperkalemia and describes the management of such cases in neonates.

**Learning points:**

- PHA1 may present with a life-threatening arrhythmia.
- Presentation of PHA can be confused with congenital adrenal hyperplasia.
- Timing and appropriate medical management in the critical care unit prevented fatality from severe neonatal PHA.

## Background

The causes of hyperkalemia in infancy include acute hemolysis, kidney disorders, and hormonal disorders. Common hormonal disorders leading to hyperkalemia in neonate and infants include congenital adrenal hyperplasia (CAH) and adrenal insufficiency while pseudohypoaldosteronism (PHA) remains a rare cause. PHA can result in severe hyponatremia, hyperkalemia, and metabolic acidosis, which if undetected and untreated may cause neonatal mortality. Aldosterone is the primary hormone involved in the regulation of sodium and potassium. In the distal tubules aldosterone acts on the mineralocorticoid receptor (MR) located intracellularly and upon diffusion into the cell it makes a aldosterone mineralocorticoid receptor complex. This leads its translocation in nucleus and activates transcriptional machinery. As such, the transcription into mRNA of the DNA sequence in the activated gene regulates sodium reabsorption and potassium excretion (1). Most patients with PHA1 have identifiable mutations, but pathogenesis of PHA1 still remains elusive (2).

Herein, we describe the clinical presentation, diagnosis, management of PHA1, and review of literature. Our goal is that physicians will recognize this entity and provide judicious management.

## Case presentation

A 10-day-old female baby was born at term with a spontaneous delivery without any complications. Mother was G2, P1; her first pregnancy was a loss resulting from an ectopic pregnancy. The birth weight of baby was 8 lb. Both prenatal and post-natal courses were normal. The infant was fed with breast milk for 10–15 min from each breast every 2–3 h. However, the patient was observed by her pediatrician and noted to be underweight during the well-baby checkup at day 7 after birth. At that time, the mother complained that the baby was feeding poorly; however, on physical examination there were no signs of virilization and the external genitalia was normal for a female. After three days, she presented to the emergency department with difficulty in breathing and lethargy. Her documented weight in the emergency room was 7 lb. The patient was noted to have bradycardia, heart rate 63/min, and blood pressure of 41/30 mmHg. She was given epinephrine and placed on a dopamine drip. Then she was intubated and an ECG revealed elevated T wave and a wide QRS complex. With regard to her family history, the patient has no siblings. The parents are separated and her father lives in a different country. The mother states that the baby's father had episodes of dehydration and liked eating salt; however, the father is healthy and has no obvious medical problem to her knowledge. The mother remains completely asymptomatic.

## Investigation

Her electrolytes showed serum potassium of >9 mEq/dl, sodium 117 mEq/l, bicarbonate 7 mEq/dl, BUN 61 mg/dl, and creatinine 1.1 mg/dl. Ammonia level was noted to be 89 μg/dl (normal range, 15–45 μg/dl) and lactate level was 11.1 mmol/l (normal range, 0.5–5 mmol/l). Hemogram showed a hemoglobin content of 17.2, HCT 49.5, WBC 25.4, neutrophils 58%, bands 7%, lymphocytes 79, and platelets 394K. Blood, urine, and spinal fluid cultures were obtained.

Laboratory data revealed elevated levels of aldosterone of 660 ng/ml (normal range, 6–180 ng/dl) and renin plasma activity of 58 ng/ml per h (normal range, 2–10.2 ng/ml per h). Transtubular K gradient was 5, with normal urine osmolality indicating the aldosterone resistance. Deoxycorticosterone, deoxycortisol, androstenedione, specific S, DHEA, DOC, 17-OH-pregnenolone, progesterone, 17-α-OH-progesterone, testosterone, and adrenocorticotropic hormone (ACTH) levels were obtained to rule out CAH and all results were normal. Her 0800 h baseline serum cortisol level after low-dose ACTH stimulation was 20 μg/dl (normal range, 5–23 μg/dl). Newborn screening was negative for CAH. Patient's renal ultrasound was normal and pelvic ultrasound revealed a normal-sized uterus but the ovaries could not be visualized due to technical difficulty. Her karyotype revealed a normal female chromosome 46,XX. Sweat test at 1 year of age was <25 mmol/l (normal, <39 mmol/l for age). The post-intervention chest X-ray and echocardiogram obtained were normal.

## Treatment

The baby was empirically treated with vancomycin and cefotaxime for presumed sepsis. i.v. sodium chloride, sodium bicarbonate, i.v. calcium, insulin and dextrose, albuterol, and rectal kayexalate were administered for volume repletion and potassium stabilization.

On day four, the baby was receiving 12 mEq/kg per day sodium chloride and 6 g kayexalate/day. She continued to improve and was extubated on day 5 of hospitalization. She was given oral sodium chloride and sodium bicarbonate along with rectal kayexalate. The infant was discharged after 30 days of hospitalization with 3 mEq/kg per day sodium chloride, 1.5 g kayexalate/kg per day, and 2 mEq/kg per day sodium bicarbonate. Spot urine electrolytes revealed urine sodium of 140 mmol/l (H) and potassium of 3 mmol/l (L).

## Outcome and follow-up

At the time of this report, the patient is 2 years old and she is receiving low-dose sodium chloride, sodium bicarbonate (Na 2 mEq/kg per day), and 0.2 g kayexalate/kg per day. A G-tube was placed at the age of 12 months because of the patient's failure to gain weight and regurgitation of the medications due to distaste. The patient continues to maintain linear weight and height at 10th percentile. Her developmental milestones have since improved to normal for age. The patient had two hospitalizations prior to 6 months of age for vomiting and hyperkalemia and two other visits to the emergency room for ear infection and upper respiratory infections.

## Discussion

PHA type 1 (PHA1) is a rare disorder that causes severe hyponatremia, metabolic acidosis, and life-threatening hyperkalemia with normal glomerular filtration rate (GFR) (calculated as Schwartz) formula: height (cm)×0.45/serum creatinine) in early life despite high plasma levels of aldosterone due to defects in sodium transport in the distal tubule. More than 50 mutations have been identified in the *NR3C2* gene, which encodes for MR. Loss of function leads to the phenotype of PHA1. Whereas gain to function mutation in renal MR leads to early-onset hypertension (3) (4).

Zennaro *et al*. (1) have described the first case of a newborn with severe recessive PHA1 caused by two heterozygous mutations in *NR3C2* gene coding for MR. Their conclusion was that mutation in *NR3C2* allele may be more common in cases with detected PHA1 (5). A further study was performed in Japan involving six Japanese patients with renal PHA1. The study found that there were two patients in their population who required 9 g salt/day after 1 year. No mutation was reported in their families, which suggests *de novo* mutation. Therefore, these studies show that renal PHA1 has a broad phenotypic range (6). Some patients with renal PHA1 could even require salt supplementation into the second year of life.

Apart from an autosomal dominant or renal PHA1 form, other forms of PHA also include an autosomal recessive or systemic PHA1 form and a transient form or PHA3. Renal PHA1 is caused by defects in the MR located on chromosome 4q31.1–31.2. This receptor is activated by aldosterone and glucocorticoids. Activation of receptor occurs after ligand binding of aldosterone resulting in transduction with specific expression in nucleus, which in turn results in transcription in mRNA of the DNA sequence of the activated gene (2).

Patients diagnosed with renal PHA have a good prognosis and their symptoms tend to spontaneously resolve between the ages of 1 and 3. Though these patients appear to be phenotypically asymptomatic, their symptoms may resurface and may have abnormal electrolytes following a period of dietary salt restriction or acute episode of dehydration even in late age. In normal physiology when systemic sodium levels drop, renin is secreted by juxtaglomerular cells located in the distal convoluted tubule. Renin in turn stimulates the secretion of aldosterone from the adrenal glands. Aldosterone then acts on MR to increase the reabsorption of sodium through the nephrons. As PHA1 is attributed to the mutation of MR, these patients present with severe urinary salt wasting, volume contraction, metabolic acidosis, and hyperkalemia leading to arrhythmias.

Systemic PHA1B or autosomal recessive form results from homozygous or compound heterozygous mutations in *SCNN1A*, *SCNN1B*, or *SCNN1G* genes. These three genes encode for subunits of the protein complex of epithelial sodium channel (ENaC): the α, β, and the γ subunits respectively. ENaC subunits form the sodium channel, which is essential for sodium and potassium transport. PHA1B is much more severe as it affects not only the kidneys but also the salivary glands, colon, respiratory tract, and sweat glands. PHA1B can be characterized by very severe salt wasting requiring lifelong management and carries a very poor prognosis. These patients have increased propensity for lung and skin infections, thus requiring frequent hospitalizations. They also tend to have frequent episodes of diarrhea and inability to gain weight.

Transient PHA or PHA3 is commonly seen with urinary tract infection (UTI) and obstructive uropathy. It may also be seen with gastrointestinal surgical problems (7) (8). Commonly, these cases present with low GFR and electrolytes normalize after treatment.

At presentation, PHA may be over shadowed by the diagnosis of 21-hydroxylase-deficient CAH, which presents with similar electrolyte imbalance along with hypoglycemia. Our patient did not have virilization, and normal 17-α-OHP level and chromosomal study revealed 46,XX female (9). Newborn screening for CAH and 21-hydroxylase genetic testing was also normal.

All patients suspected of PHA or CAH should get renal and pelvic ultrasound to visualize renal obstructive and pelvic anomalies. Our patient's abdominal ultrasound was normal; however, the pelvic ultrasound could not provide us the needed information due to technical difficulty.

Interestingly, asymptomatic parents of children with PHA1 have been found to have high aldosterone level and plasma renin activity. Therefore, obtaining serum aldosterone level and plasma renin activity in asymptomatic parents can further substantiate the diagnosis of renal PHA. In our case, the mother refused to be tested. We recommend that serum aldosterone level and plasma renin activity should be measured in parents (10).

The main stay of treatment of PHA1 includes sodium chloride and bicarbonate supplements and kayexalate. Patients receiving rectal administration of kayexalate should be closely monitored due to high incidence of bowel perforation. Both forms of PHA1 are nonresponsive to mineralocorticoids. Our patient received steroids at the time of presentation with no biochemical benefit and was discontinued once the diagnosis of PHA was confirmed. A brief trial of mineralocorticoids was ineffective in normalizing the electrolytes and was discontinued. Invariably due to failure to gain weight and dislike of oral administration of medications, G-tube placement is strongly advocated.

Our case presented with severe hyponatremia, hyperkalemia, metabolic acidosis, and weight loss at seven days of life. Severe hyperkalemia led to arrhythmia and cardiorespiratory arrest. Aldosterone level and plasma renin activity were elevated, while cortisol concentrations were normal consistent with the diagnosis of PHA1. Timing and appropriate medical management in the critical care unit prevented fatality from severe neonatal PHA1. Following acute care management, the patient was started on oral sodium chloride, sodium bicarbonate, and kayexalate. The patient is 2 years old at the time of this report and continues on low-dose sodium chloride and bicarbonate, and small dose of kayexalate. On clinical grounds, our case is consistent with PHA1; molecular studies may have helped confirming the genetic origin in this patient but were not obtained. Furthermore, according to the available clinical data, the link between genotype and phenotype in MR mutation remains elusive (11).

The infant had a hospital admission due to hyperkalemia secondary to dehydration but has had no skin or pulmonary involvement. Riepe *et al*. (12) has reported that some patients with PHA1 need higher sodium supplementation for a relatively long time ranging up to 1–3 years. Our patient's father has episodes of dehydration and salt craving but normal growth and no hospitalization. As proposed by Geller *et al*. (13), these distinct differences in disease severity between various members within one family carrying the same mutation can be observed. Our patient continues normal linear growth and height in 10th percentile with normal electrolytes and her sodium requirement is consistently decreasing. She had two hospitalizations prior to 6 months of age for hyperkalemia.

These children should be followed by a multidisciplinary team including: a dietician, social worker, endocrinologist, and neurologist. Initially, electrolytes should be monitored closely. Parents should be instructed to contact a physician when the child has vomiting or diarrhea. Parents should be informed and compliant to avoid life-threatening complications including mortality. Dieticians should advise a low-potassium and high-sodium diet. As these children have poor growth, a high-calorie diet is advised, preferably through a G-tube (14). Though majority of renal PHA1 cases are due to heterozygous mutations in *NR3C2*, 30% of cases have not shown mutation in the *NR3C2* gene. A number of likely possibilities, namely presence of a different gene causing electrolyte imbalance resembling PHA or environmental factors in the pathogenesis of PHA1, remain open. In a study, patients with PHA1 underwent cardiac, vascular ultrasound, and cardiac magnetic resonance imaging to document the long-term cardiovascular outcomes as adults. When compared with the noncarriers of the same gender and age, the patients with PHA1 had improved diastolic left ventricular function without any adverse outcomes, suggesting that adulthood mutation in the MR offers cardioprotection from the effects of elevated aldosterone levels (15).

In conclusion, PHA1 caused by defects in the MR is a rare disorder that causes severe hyponatremia, metabolic acidosis, and life-threatening hyperkalemia, with normal 17-α-hydroxyprogesterone (17-α-OHP) levels and high plasma levels of aldosterone and PRA levels. Clinicians treating neonates presenting with hyperkalemia, hyponatremia, and metabolic acidosis should always consider urinary tract infection and obstructive uropathy; physicians should be aware that, in PHA1, electrolyte derangements are severe and persistent, while the GFR is normal. Once diagnosed, treatment should include aggressive i.v. hydration with sodium chloride, sodium bicarbonate supplementation, and correction of hyperkalemia. Laboratory investigation should include serum 17-α-OHP, aldosterone, cortisol, and plasma renin activity. In cases of refractory hyperkalemia, peritoneal dialysis should be quickly instituted. A majority of children with PHA1 need salt replacement until the first year of life, but some such as the one described here require salt supplementation well until they are 2–3 years old. A close outpatient follow-up with a multidisciplinary team is required. PHA can be diagnosed clinically and one should not wait for genetic testing. The relationship of mutation and phenotype still remains elusive. Future research is needed to enhance our understanding of this complex disease.

## Patient consent

We have obtained the informed consent from the patient's mother, and the Institutional Committee of Human Research approves this report.

## Author contribution statement

S K Rajpoot has significantly contributed to the write-up of report. A Bhangoo has contributed to the biochemical analysis of endocrine function in the laboratory and write-up of the report. C Maggi is the physician responsible for the care of the patient
